# GPR87 promotes tumor cell invasion and mediates the immunogenomic landscape of lung adenocarcinoma

**DOI:** 10.1038/s42003-022-03506-6

**Published:** 2022-07-05

**Authors:** Rui Bai, Jianguo Zhang, Fajian He, Yangyi Li, Panpan Dai, Zhengrong Huang, Linzhi Han, Zhihao Wang, Yan Gong, Conghua Xie

**Affiliations:** 1 Department of Radiation and Medical Oncology, Zhongnan Hospital of Wuhan University, Wuhan, Hubei 430071 China; 2 Department of Biological Repositories, Zhongnan Hospital of Wuhan University, Wuhan, Hubei 430071 China; 3 Tumor Precision Diagnosis and Treatment Technology and Translational Medicine, Hubei Engineering Research Center, Zhongnan Hospital of Wuhan University, Wuhan, Hubei 430071 China; 4 Hubei Key Laboratory of Tumour Biological Behaviors, Zhongnan Hospital of Wuhan University, Wuhan, Hubei 430071 China

**Keywords:** Cancer, Immunology

## Abstract

The purpose of this study is to examine the association between G protein-coupled receptor 87 (GPR87) and lung adenocarcinoma (LUAD) metastasis and immune infiltration. The Cancer Genome Atlas (TCGA) and Gene Expression Omnibus (GEO) datasets extract clinical data. According to the TCGA database, increased *GPR87* expression predicts poor overall survival, progression-free interval, and disease-specific survival in LUAD patients. The meta-analysis also reveals a significant association between high *GPR87* expression and poor overall survival. Moreover, functional experiments demonstrate that *GPR87* silencing reduces LUAD cell invasion and migration. Immunoblotting shows that GPR87 knockdown decreased Vimentin and N-cadherin expression and increased E-cadherin expression in LUAD cells. *GPR87* expression in LUAD is positively correlated with immune infiltration. In addition, *GPR87* expression is associated with immune and chemotherapy resistance in LUAD patients. Our findings indicate that *GPR87* promotes tumor progression and is correlated with immune infiltration, suggesting *GPR87* as a possible biomarker for prognosis prediction in LUAD.

## Abstract (web summary)

GPR87 is reported as a central player in lung adenocarcinoma and in resistance to immunotherapy, by promoting tumor cell invasion and mediating the immunogenomic landscape.

## Introduction

With an estimated 1.6 million new cases and 1.38 million deaths each year, lung cancer is the leading cause of cancer death globally^1^. Lung adenocarcinoma (LUAD) as the most prevalent subtype, accounts for 40% of non-small cell lung cancer (NSCLC) at diagnosis^2^. Although treatments such as chemotherapy, radiotherapy, and immunotherapy have been improved over the previous decades, the overall survival (OS) of LUAD patients remains low^3^. Thus, it is critical to identify prognostic indicators and treatment targets.

The epithelial-mesenchymal transition (EMT) is a critical cellular process controlled by a series of EMT-inducing transcription factors. It is characterized by the loss of epithelial characteristics and the acquisition of mesenchymal markers^4^. Not only is EMT related to tumor growth, but it is also associated with poor prognosis and medication resistance in LUAD^5^. Analysis of RNA expression profiles to identify therapeutic target genes has become a hot issue in recent years to improve prognosis^6^. Therefore, finding prognostic genes associated with EMT by sequencing may help improve LUAD patients’ prognoses.

G protein-coupled receptors comprise about 800 proteins, as the most prominent family of eukaryotic membrane signaling proteins^7^. G protein-coupled receptor (GPR) 87 locates at chromosome 3q24 and encodes a protein with an extracellular N-terminal, seven helixes, and three intracellularly loops^8^. It is overexpressed on the cell surface in different malignancies and is crucial to the survival of tumor cells. Zhang et al. found that *GPR87* has an important role in the response of p53-dependent cells to DNA damage. By enhancing the stabilization and activation of p53, inhibition of *GPR87* expression could sensitize cancer cells to growth inhibition induced by DNA damage^9^. *GPR87* is highly expressed in NSCLC and associated with cell proliferation^10–12^. However, its EMT and immune function is to be investigated.

We examined the differential expression of *GPR87* in LUAD and normal tissues and the relationship between its gene expression and DNA methylation in TCGA. Additionally, we assessed the predictive values of *GPR87* expression and methylation. The predictive effects of *GPR87* were then verified using 4 independent GEO datasets. A complete meta-analysis was conducted using data from five public databases to determine the overall prognostic importance of *GPR87*. Finally, in vitro experiments confirmed that GPR87 downregulation hindered the EMT process of LUAD cells. In conclusion, our findings revealed the regulatory effects and the underlying mechanisms of *GPR87* on tumor metastasis in LUAD, implying that *GPR87* might be an important prognostic and therapeutic target for LUAD patients.

## Results

### *GPR87* is highly expressed in LUAD and associated with lymph node metastasis

**Fig. 1:**
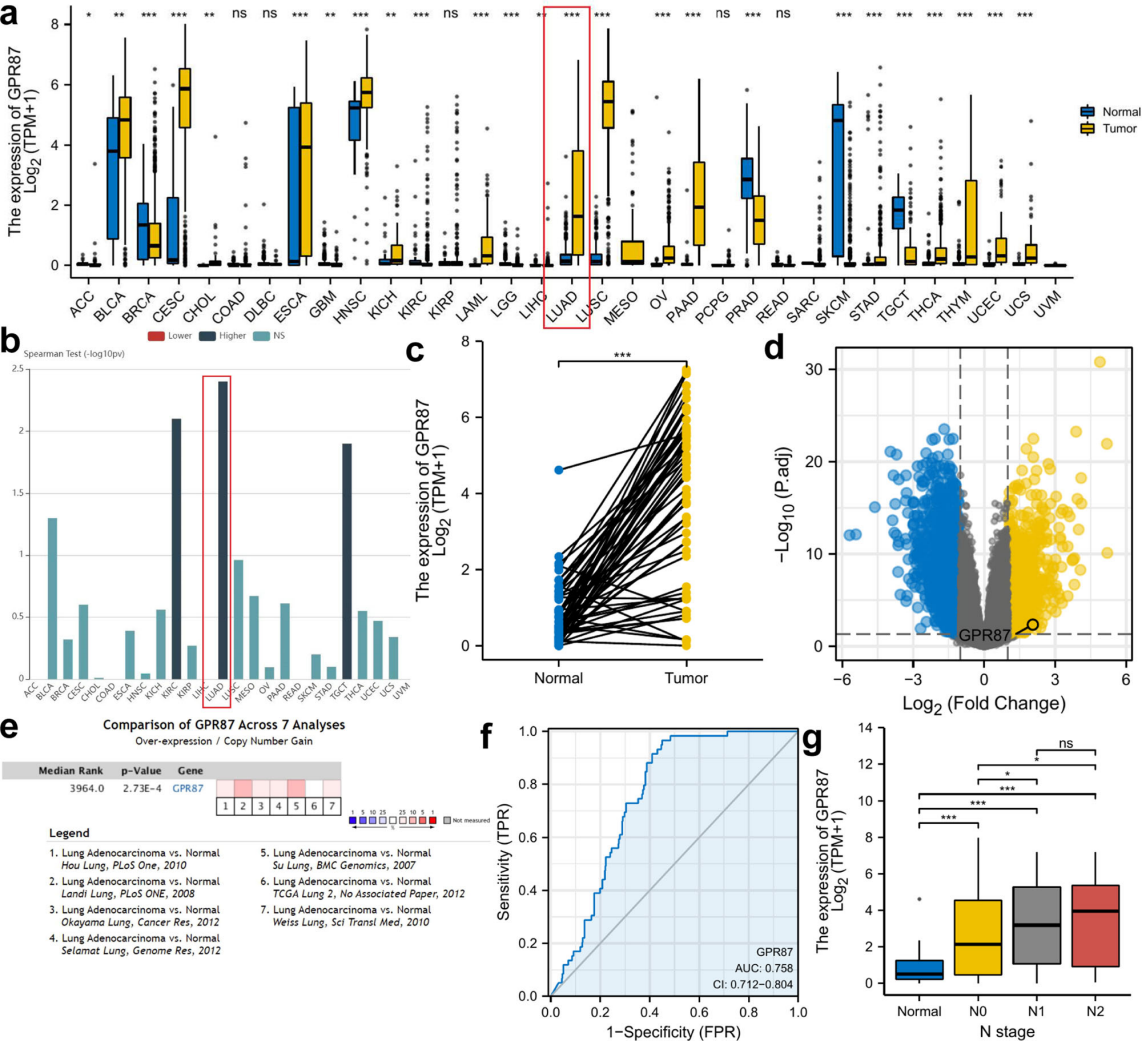
*GPR87* expression in LUAD and adjacent normal tissues. **a** Expression of the *GPR87* gene in pan-cancer using TCGA data. **b** The relationship between *GPR87* and the clinical stages of all TCGA cancers using TISIDB. **c** *GPR87* expression levels in 57 paired normal and tumor and tissues. **d** Volcano plot of differential analysis in GSE31210. **e** Obtaining differential expression of *GPR87* in lung cancer using Oncomine database. **f** ROC curve to verify the accuracy of *GPR87* to distinguish tumor and normal tissues. **g** Detection of *GPR87* expression levels concerning tumor lymph node metastasis.

First, we assessed the distribution of *GPR87* expression in all tumor tissues and found that *GPR87* expression was elevated in most cancers (Fig. 1a). Next, we examined the relationship between *GPR87* and the clinical stages of all TCGA cancers using TISIDB (Fig. 1b). *GPR87* mRNA levels were most significantly correlated with the clinical stages of LUAD patients. Therefore, we focused on the role of *GPR87* in LUAD progression. To validate the high expression of *GPR87* in LUAD, we examined *GPR87* expression in 57 paired LUAD patients from the GEO dataset GSE31210, Oncomine, and TCGA (Fig. 1c–e). We confirmed that the expression of *GPR87* was significantly higher in LUAD than in normal tissues. Meanwhile, *GPR87* was moderately accurate in predicting tumor and normal outcomes (Fig. 1f, AUC = 0.758, CI = 0.712–0.804). To investigate the relationship between *GPR87* expression and lymph node metastasis, we investigated the breakdown of *GPR87* expression by N stage in LUAD. High *GPR87* levels in LUAD were also associated with a high grade of lymph node metastasis (Fig. 1g).

### Functional enrichment analysis shows that *GPR87* is associated with EMT

**Fig. 2:**
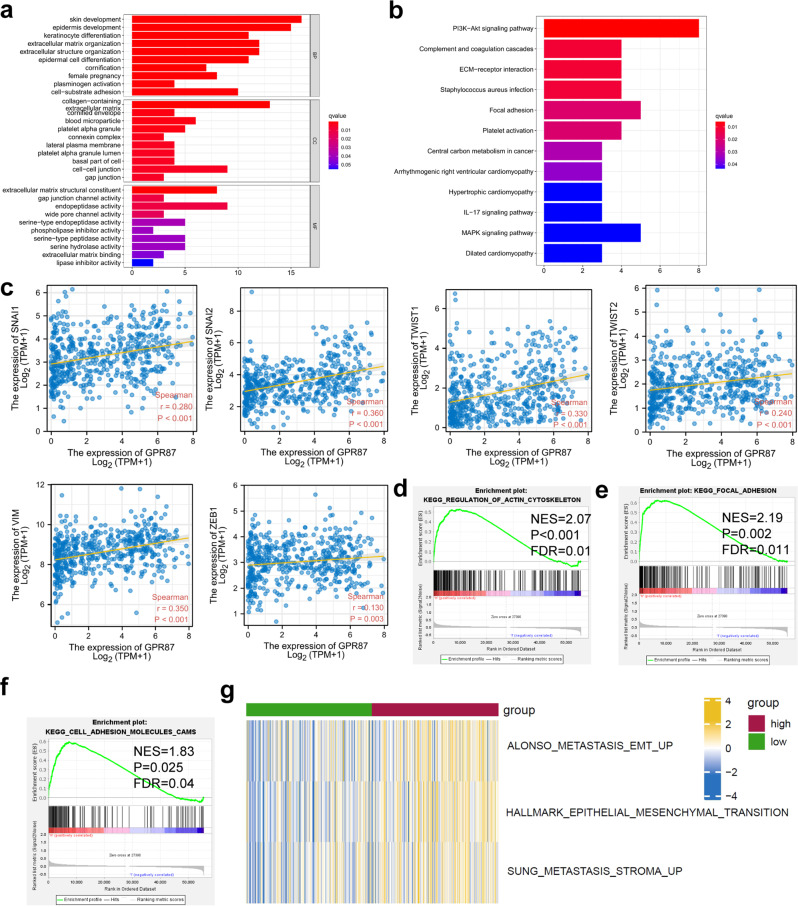
Functional enrichment analysis to verify the association of *GPR87* with EMT. **a** GO enrichment analysis. **b** KEGG enrichment analysis. **c** *GPR87* is positively correlated with EMT-related gene transcription. Regulation of actin cytoskeleton (**d**), focal adhesion (**e**), and CAMs (**f**). **g** RNA-seq data from LUAD patients in the TCGA database were analyzed using the ssGSEA algorithm to evaluate the association of *GPR87* with tumor metastasis.

To verify whether the *GPR87* was correlated with the EMT process, we first separated the patients into high- and low-*GPR87* expressing groups based on the median *GPR87* mRNA level. Differential expression analysis was performed in these two groups using limma. Differentially expressed genes (DEGs) were then integrated into the subsequent GO and KEGG analysis, and the results showed that *GPR87* was associated with a variety of EMT-related functions, including cell-cell junction, gap junction, and Focal adhesion (Fig. 2a, b). The results of the KEGG enrichment analysis showed that *GPR87* was positively correlated with the transcription of SNAI1, SNAI2, TWIST1, TWIST2, VIM, and ZEB1, which were EMT-associated transcription factors (Fig. 2c). We further performed enrichment analysis using GSEA and found that *GPR87* was also associated with cell adhesion molecules (CAMs), focal adhesion, and regulation of actin cytoskeleton, further confirming the involvement of *GPR87* in the regulation of EMT (Fig. 2d–f). In addition, we also scored the EMT of each LUAD patient in TCGA using the Single-sample gene set enrichment analysis (ssGSEA) algorithm. The results showed that the metastasis-related scores were significantly higher in the high *GPR87* expressing group (Fig. 2g).

### *GPR87* plays an oncogene role in LUAD

**Fig. 3:**
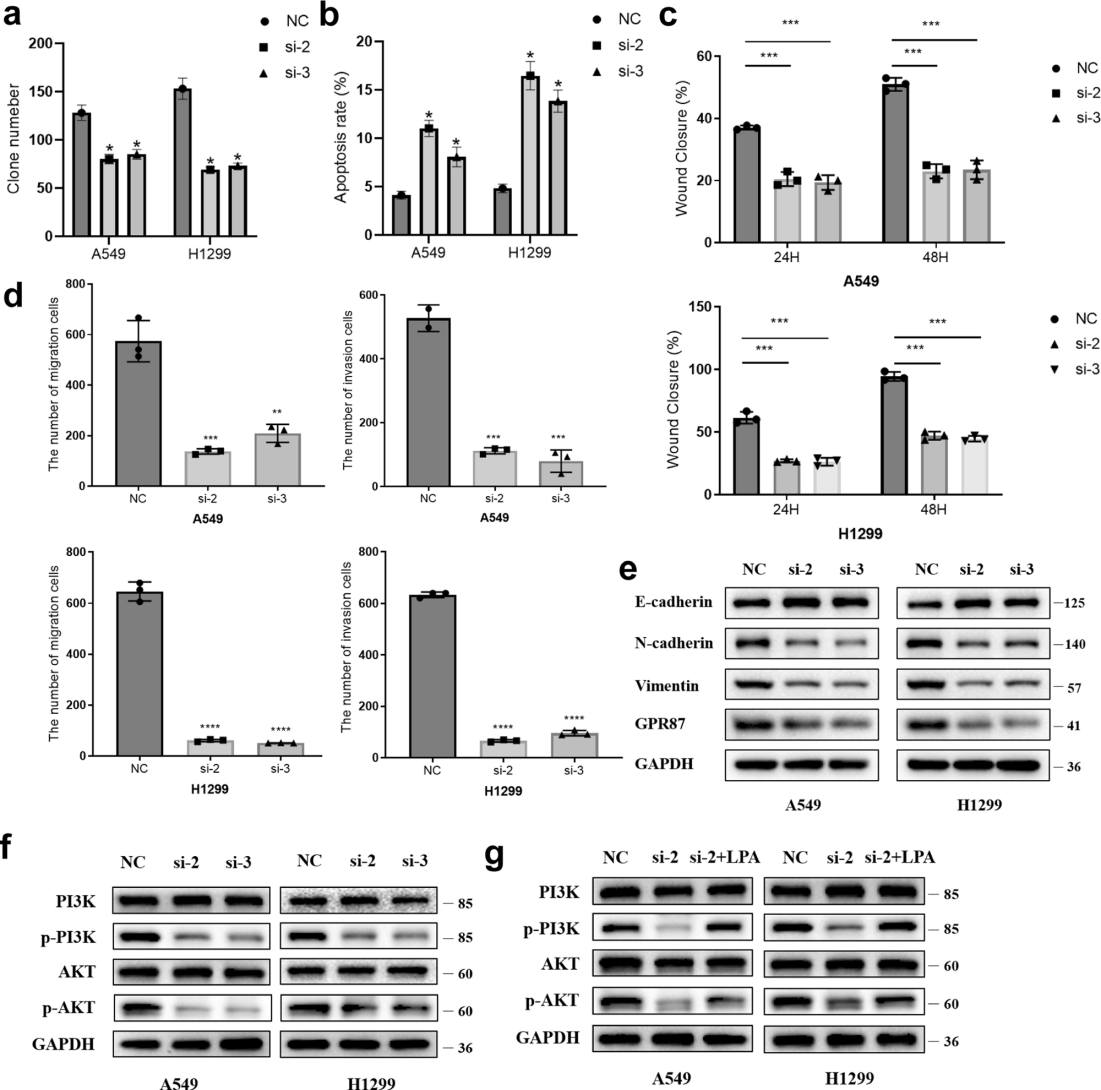
GPR87 knockdown inhibits LUAD cell clonogenesis, apoptosis, and EMT. **a** Quantitative results of clone formation experiments. **b** Quantitative results of cell apoptosis experiments. **c** Cell migration was evaluated by wound healing assay and reduced by GPR87 knockdown. Quantification of relative wound closure. **d** Migration and invasion of A549 and H1299 cells transfected with GPR87 siRNA were evaluated by a modified Boyden chamber assay. Quantification of the migration and invasion cells. **e** Representative immunoblotting of EMT-related proteins. **f** Representative immunoblotting of PI3K signaling pathway-related proteins. **g** After the knockdown of GPR87, the LPA receptor (5 µM) was added to the culture medium and WB assayed PI3K signaling pathway activation.

To investigate the effects of *GPR87* knockdown on EMT in LUAD cells, *GPR87* siRNA was transfected into A549 and H1299 cells. The results of qRT-PCR showed that *GPR87* mRNA expression was significantly reduced after siRNA transfection (Fig. S1). Immunoblotting confirmed that GPR87 protein was downregulated in both A549 and H1299 cells (Fig. 3e). The results of the clone formation assay showed that knockdown of GPR87 significantly inhibited the proliferation of A549 and H1299 cells (Fig. 3a and Fig. S2a). Flow cytometry assay of apoptosis showed that knockdown of GPR87 significantly promoted the apoptosis rate of A549 and H1299 cells (Fig. 3b and Fig. S2b). Wound healing and modified Boyden chamber assay were used to examine the effects of GPR87 knockdown on the migration and invasion of LUAD cells. The migration and invasion rates of A549-KD and H1299-KD cells were significantly lower than NC ones (Fig. 3c, d and Fig. S2c, d). Immunoblotting confirmed the elevated levels of the epithelial marker E-cadherin. GPR87 knockdown decreased the levels of mesenchymal markers, such as Vimentin and N-cadherin (Fig. 3e). According to the results of the KEGG enrichment analysis, *GPR87* is highly correlated with the PI3K-Akt signaling pathway, so we examined the activation of the PI3K-Akt pathway after the knockdown of GPR87 by immunoblotting, and the results showed that inhibition of GPR87 inhibited the activation of PI3K-Akt pathway (Fig. 3f). LPA, as a ligand of GPR87, could partially reverse the inhibition of the PI3K-Akt pathway caused by the knockdown of GPR87 (Fig. 3g). These results suggested that GPR87 knockdown induced the transition to epithelial phenotype in A549 and H1299 cells.

### *GPR87* is associated with a poor prognosis

**Fig. 4:**
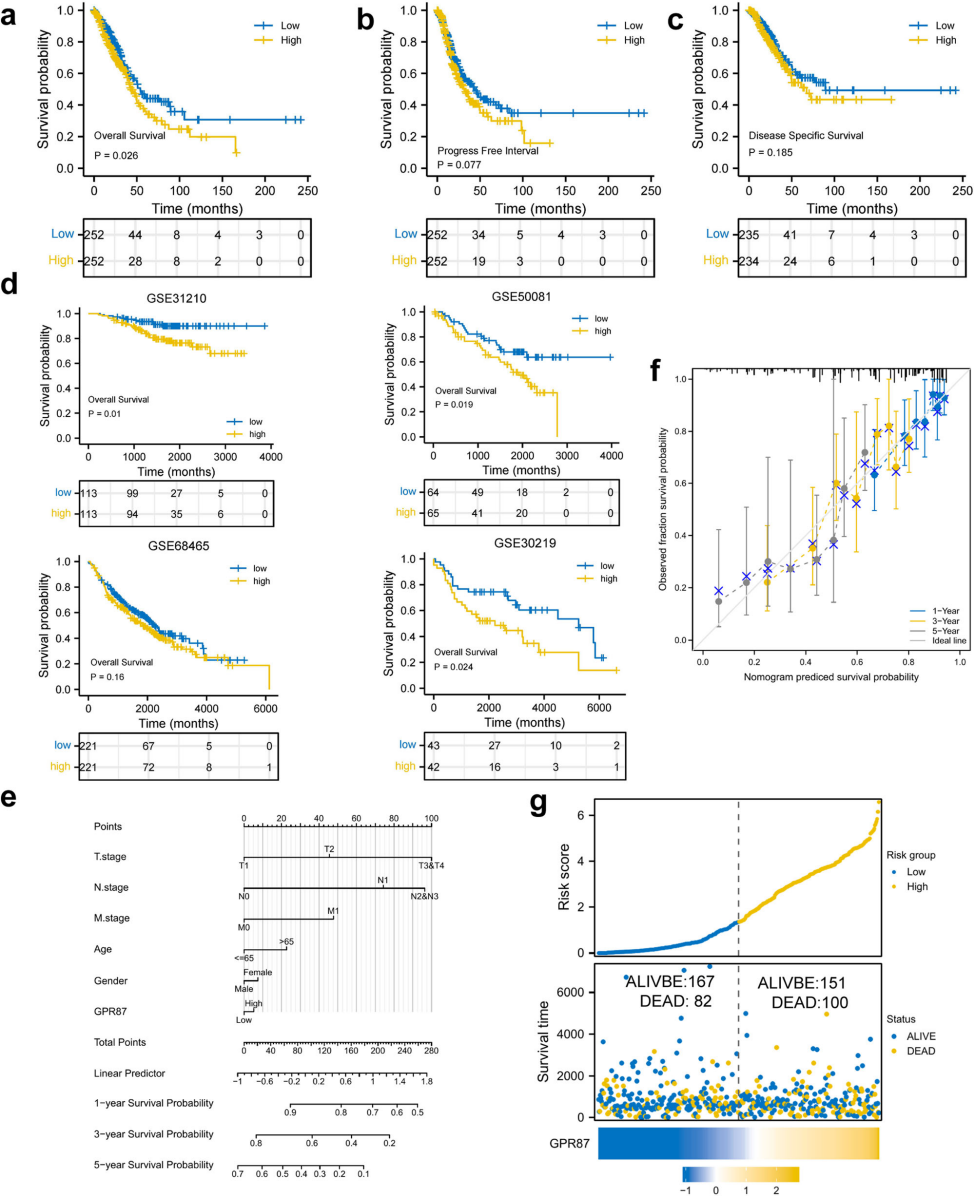
Kaplan–Meier curves of GPR87 in LUAD patients. OS (**a**), disease-specific survival (**c**), and progress-free interval (**b**) in the TCGA-LUAD dataset. **d** OS in the GSE31210, GSE50081, GSE68465, and GSE30219. **e** *GPR87* was combined with other clinical factors to plot the nomogram and predict the prognosis of LUAD patients. **f** The calibration curve was drawn to verify the accuracy of the prediction model in predicting 1-, 3-, and 5-year survival rates. **g** Survival conditions of LUAD patients.

We further analyzed the association between *GPR87* and different clinical outcomes in LUAD patients. Survival analysis showed that the high-expression group was associated with shorter OS (HR = 1.39, 95% CI [1.04−1.86], *P* < 0.05), disease-specific survival (HR = 1.29, 95% CI [0.89−1.87], *P* = 0.185), and progress-free interval (HR = 1.28, 95% CI [0.97−1.69], *P* = 0.077, Fig. 4a–c). In addition to the TCGA data, four independent datasets were selected as the validation sets to confirm the effects of *GPR87* on the prognosis of LUAD patients. The results of these independent datasets were consistent with the TCGA results, and *GPR87* expression was associated with poor prognosis (GSE30219: HR = 2.01, 95% CI [1.10−3.68], *P* < 0.05; GSE31210: HR = 2.63, 95% CI [1.26−5.49], *P* < 0.05; GSE50081: HR = 1.95, 95% CI [1.12−3.42], *P* < 0.05, GSE68465: HR = 1.2, 95% CI [0.93−1.55]) (Fig. 4d). Meanwhile, we drew a nomogram using *GPR87* expression together with clinical factors and plotted calibration curves to validate the accuracy of the prediction model (Fig. 4e, f). The predicted values matched well with the actual values, indicating that our model could be applied to predict the prognosis of LUAD patients. The survival status, survival time, and *GPR87* expression levels of the LUAD patients were shown in Fig. 4g. With the increase in the risk scores, the number of deaths also increased.

### Meta-analysis also shows the prognostic value of *GPR87* in LUAD

**Fig. 5:**
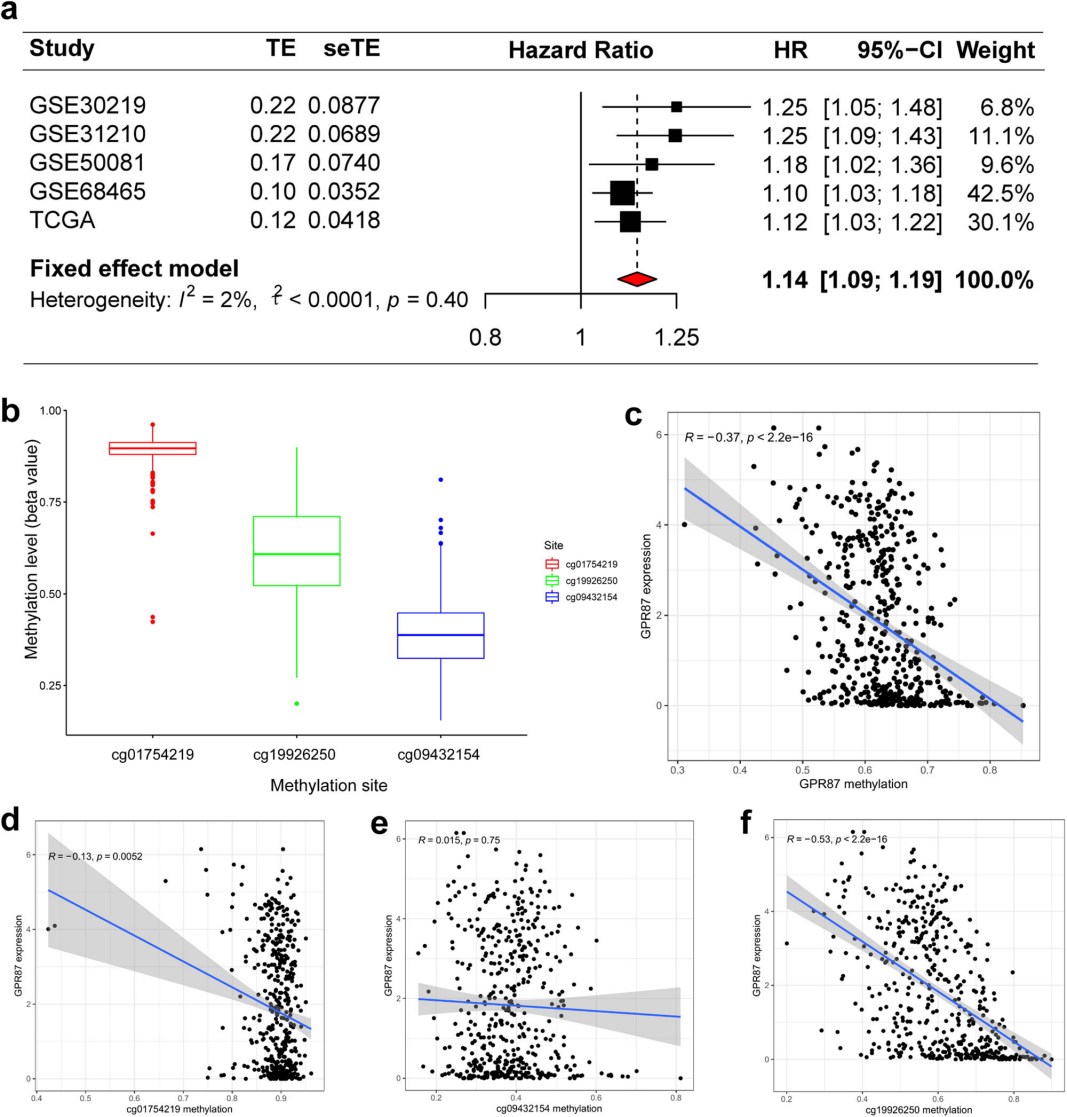
*GPR87* methylation and its effects on prognosis in LUAD tissues were revealed by bioinformatics analysis. **a** Forest plots of high *GPR87* expression from the four datasets with worse OS in LUAD patients. **b** Distribution of *GPR87* promoter CpG sites. **c** Correlation analysis between *GPR87* methylation and expression levels. **d**–**f** Correlation analysis between methylation levels of CpG sites and *GPR87* expression.

Due to few reports on the association between *GPR87* expression and OS in LUAD patients, we integrated the prognosis from the five different datasets into the meta-analysis. The combined HR and 95% CI association between *GPR87* expression and OS was 1.31 (1.22–1.40) in 1390 LUAD patients, with no significant heterogeneity between the 4 datasets (I2 = 5%, *P* = 0.37, Fig. 5a). Therefore, we concluded that high expression of *GPR87* was a robust predictor of poor prognosis in LUAD patients.

### DNA methylation of *GPR87* inhibits its expression

To investigate the relationship between *GPR87* DNA methylation and gene expression, we analyzed the *GPR87* methylation sites, which are mainly distributed in three *GPR87* CpG sites (Fig. 5b). Spearman correlation analysis was used to identify the sites that correlated with *GPR87* expression (Fig. 5c). Except cg09432154, the methylation of the other CpG sites was correlated with *GPR87* expression, and the most correlated site was cg19926250 (Fig. 5d–f). Based on the *GPR87* methylation levels, the patients were divided into the *GPR87* hypo- and hypermethylated groups. We used chi-square tests to investigate *GPR87* expression and gene methylation correlations with a range of clinical features. *GPR87* expression was significantly correlated with N stage (*P* < 0.05), and *GPR87* methylation (*P* < 0.0001, Table 1). Similarly, *GPR87* expression (*P* < 0.0001) also correlated with *GPR87* methylation levels.

**Table 1:** Correlation of *GPR87* mRNA expression and methylation with clinicopathological features in the TCGA database.

			*GPR87* expression		*P*	*GPR87* methylation		*P*
Covariates	Type	Total	High	Low		High	Low	
Age	≤65	225(48.39%)	114(49.14%)	111(47.64%)	0.7726	110(47.41%)	115(49.36%)	0.4894
	>65	221(47.53%)	110(47.41%)	111(47.64%)		110(47.41%)	111(47.64%)	
	unknown	19(4.09%)	8(3.45%)	11(4.72%)		12(5.17%)	7(3%)	
M	M0	299(64.3%)	149(64.22%)	150(64.38%)	0.9701	148(63.79%)	151(64.81%)	0.7755
	M1	19(4.09%)	10(4.31%)	9(3.86%)		11(4.74%)	8(3.43%)	
	unknown	147(31.61%)	73(31.47%)	74(31.76%)		73(31.47%)	74(31.76%)	
N	N0	306(65.81%)	140(60.34%)	166(71.24%)	0.039	155(66.81%)	151(64.81%)	0.4934
	N1	81(17.42%)	47(20.26%)	34(14.59%)		36(15.52%)	45(19.31%)	
	N2	65(13.98%)	40(17.24%)	25(10.73%)		32(13.79%)	33(14.16%)	
	N3	1(0.22%)	1(0.43%)	0(0%)		1(0.43%)	0(0%)	
	unknown	12(2.58%)	4(1.72%)	8(3.43%)		8(3.45%)	4(1.72%)	
T	T1	157(33.76%)	76(32.76%)	81(34.76%)	0.885	83(35.78%)	74(31.76%)	0.3076
	T2	247(53.12%)	122(52.59%)	125(53.65%)		120(51.72%)	127(54.51%)	
	T3	42(9.03%)	23(9.91%)	19(8.15%)		20(8.62%)	22(9.44%)	
	T4	16(3.44%)	9(3.88%)	7(3%)		6(2.59%)	10(4.29%)	
	unknown	3(0.65%)	2(0.86%)	1(0.43%)		3(1.29%)	0(0%)	
Gender	female	249(53.55%)	134(57.76%)	115(49.36%)	0.0848	128(55.17%)	121(51.93%)	0.5434
	male	216(46.45%)	98(42.24%)	118(50.64%)		104(44.83%)	112(48.07%)	
Stage	stage 1	256(55.05%)	115(49.57%)	141(60.52%)	0.0837	130(56.03%)	126(54.08%)	0.1395
	stage 2	111(23.87%)	59(25.43%)	52(22.32%)		51(21.98%)	60(25.75%)	
	stage 3	73(15.7%)	46(19.83%)	27(11.59%)		34(14.66%)	39(16.74%)	
	stage 4	20(4.3%)	10(4.31%)	10(4.29%)		12(5.17%)	8(3.43%)	
	unknown	5(1.08%)	2(0.86%)	3(1.29%)		5(2.16%)	0(0%)	
Expression	high	232(49.89%)	-	-	-	90(38.79%)	142(60.94%)	<0.0001
	low	233(50.11%)	-	-		142(61.21%)	91(39.06%)	
Methylation	high	232(49.89%)	90(38.79%)	142(60.94%)	<0.0001	-	-	-
	low	233(50.11%)	142(61.21%)	91(39.06%)		-	-	

### *GPR87* expression positively correlates with immune cell infiltration and immune checkpoint expression

**Fig. 6:**
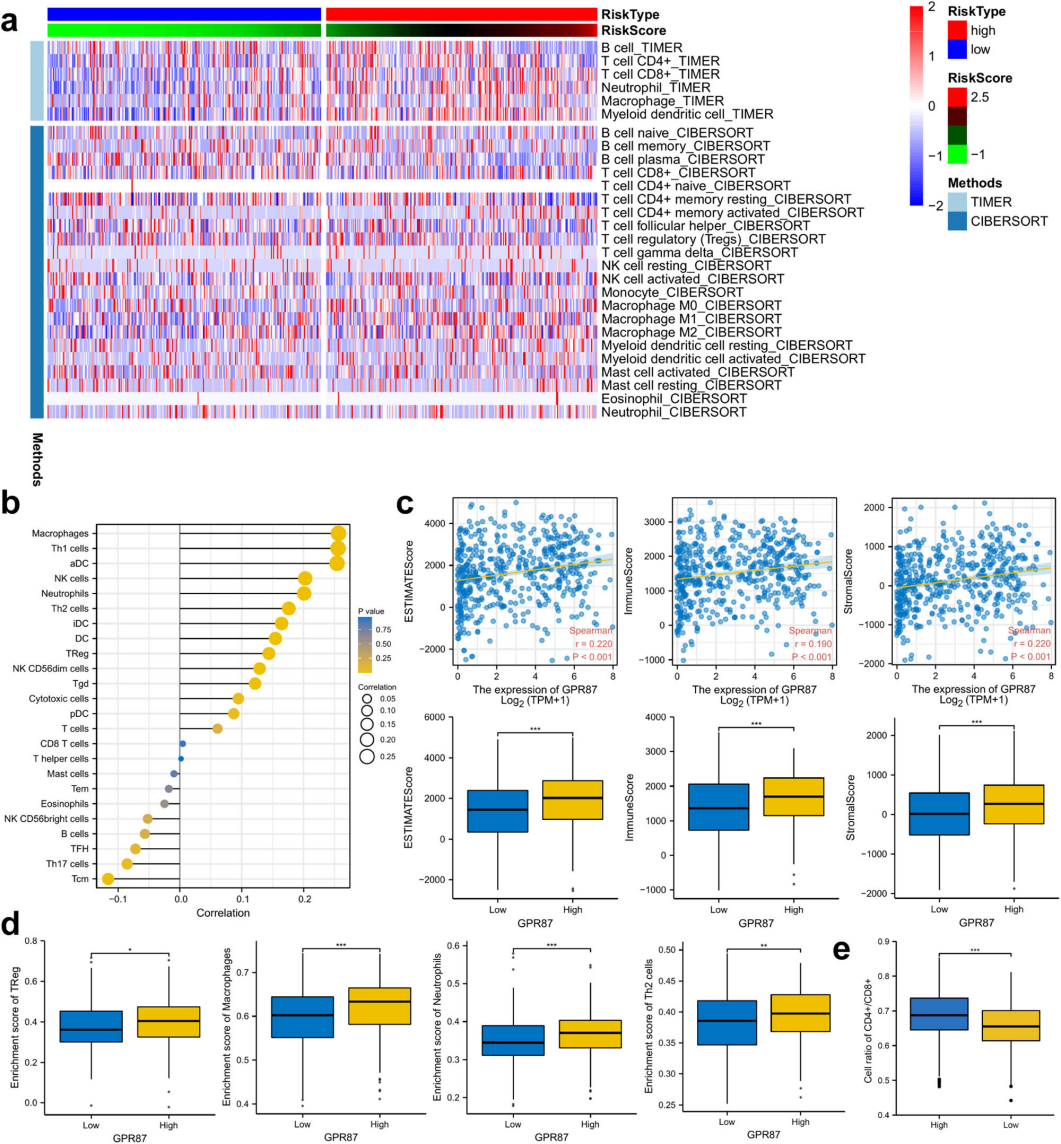
The relationship between *GPR87* expression and immune cell infiltration. **a** Heat map demonstrating the level of immune cell infiltration in patients with high and low *GPR87* expression, with immune cell infiltration evaluated by TIMER and CIBERSORT algorithms. **b** Lollipop graph shows the correlation between *GPR87* expression and immune cells. **c** *GPR87* was analyzed in correlation with immune scores and stromal scores, and differences in immune scores and stromal scores were analyzed in patients with high and low expression of *GPR87*. The immune scores and stromal scores were calculated by the ESTIMATE algorithm. **d** Differential analysis of regulatory T cells, macrophages, neutrophils, and T helper 2 cells in patients with high and low expression of *GPR87*. **e** Analysis of differences in CD4+/CD8+ cell ratios between patients with high and low expression of *GPR87*.

We used CIBERSORT and TIMER algorithm to evaluate the levels of immune cell infiltration in LUAD patients. The results showed that the levels of immune cell infiltration were higher in the *GPR87* high-expression group (Fig. 6a). *GPR87* was positively correlated with macrophages, Th1, aDC cells, etc., and negatively correlated with Tcm, Th17 cells, etc. (Fig. 6b). In addition, the immune and stromal scores of LUAD patients were calculated using the ESTIMATE algorithm, which further confirmed that patients with high expression of *GPR87* had significantly higher levels of immune infiltration than those with low expression (Fig. 6c). The result of ssGSEA showed that infiltration of regulatory T cells, macrophages, neutrophils, T helper 2 cells, and natural killer cells was significantly higher in patients with high *GPR87* expression than in patients with low expression (Fig. 6d). The proportion of CD4+/CD8+ cells was higher in patients with high *GPR87* expression (Fig. 6e).

### High *GPR87* expression promotes immunotherapy resistance and chemoresistance in LUAD

**Fig. 7:**
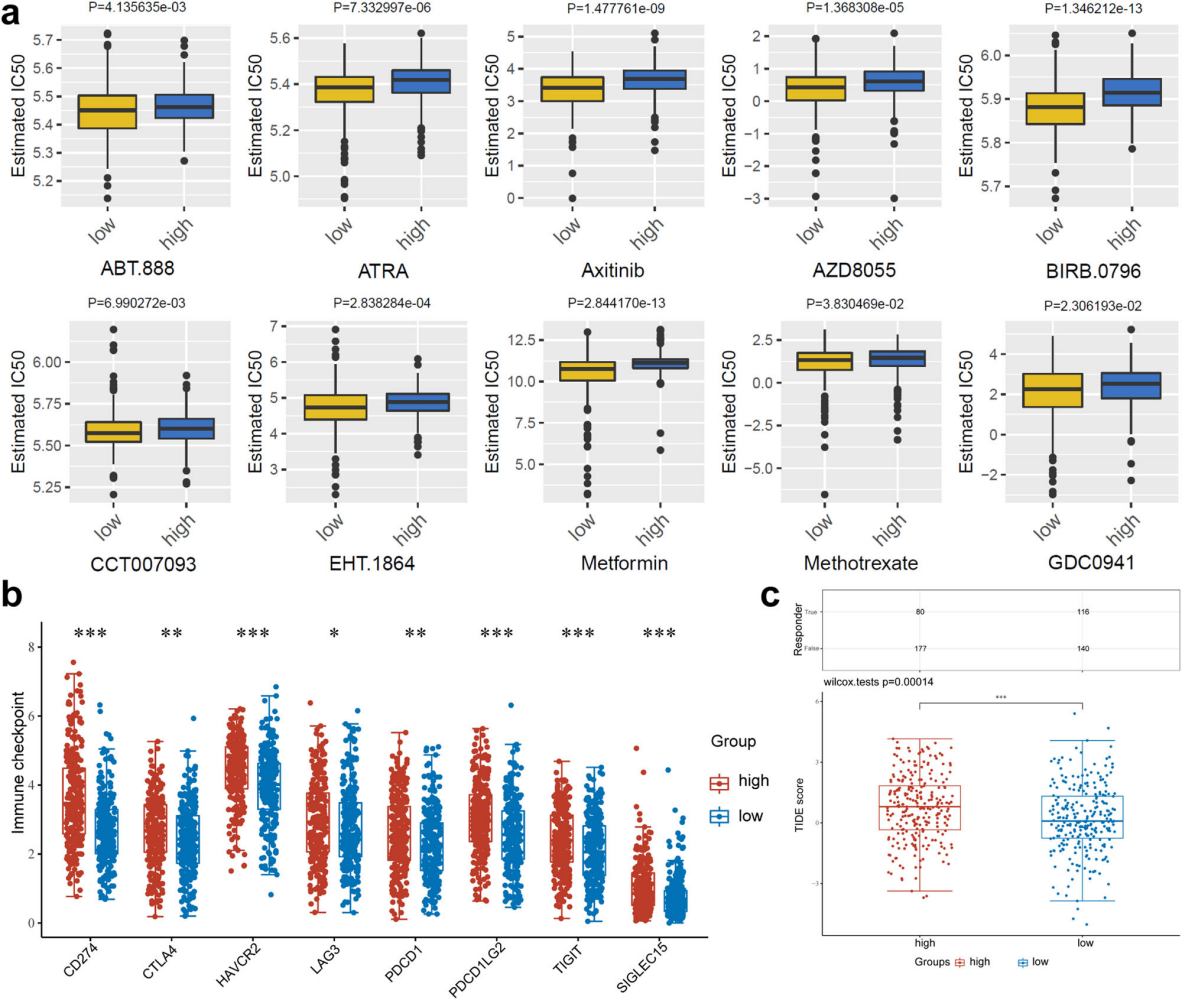
Immune infiltration and response of high- and low-*GPR87* patients to chemotherapy and immunotherapy. **a** The box plots of the estimated IC50 for ten common chemotherapeutic agents for high- and low-*GPR87* expression. **b** Distribution of immune checkpoint expression in patients with high- and low-*GPR87* expression. **c** The box plots of the TIDE scores for immunotherapy response for high- and low-*GPR87* expression.

Based on the pRRophetic package, we evaluated the effects of *GPR87* on the sensitivity of common chemotherapeutic agents. All the ten drugs (ABT.888, ATRA, axitinib, BIRB.0796, CCT007093, EHT.1864, metformin, methotrexate, GDC0941, and AZD8055) were found to have a higher estimated half-maximal inhibitory concentration (IC50) in high-risk patients (Wilcoxon test, all *P* < 0.05, Fig. 7a). Considering the vital role of immune checkpoint inhibitors (ICIs) in immunotherapy, we further investigated the differences in immune checkpoint expression between *GPR87* high- and low-expression groups. We found that CD274, HAVCR2, LAG3, PDCD1, PDCD1LG2, TIGIT, and SIGLEC15 were significantly higher in patients with high *GPR87* expression, which was consistent with the results of immune infiltration and suggested immunosuppression (Fig. 7b). This implies that LUAD patients with high *GPR87* expression may be resistant to treatment with these ten drugs, but this conclusion needs to be verified in subsequent clinical trials. We used the tumor immune dysfunction and exclusion (TIDE) algorithm to assess the effects of *GPR87* on the response rates to immune checkpoint (PD-1 and CTLA-4) inhibitors. Patients with higher TIDE scores were more likely to have tumor immune escape. Meanwhile, the TIDE score was more accurate than the PD-L1 expression levels and tumor mutation burden (TMB) to predict the survival of cancer patients treated with ICIs. TIDE scores were significantly lower in the high-*GPR87* patients than in low-*GPR87* ones (Wilcoxon test, *P* < 0.001, Fig. 7c), suggesting that high-*GPR87* patients might respond poorly to immune checkpoint inhibitors and have bad outcomes.

## Discussion

In the last decades, the importance of DEGs in tumor progression has been recognized. However, genome-wide analysis remains to be investigated to explore its molecular mechanisms and clinical significance. NSCLC is one of the most common malignancies globally, and early diagnosis and treatment of NSCLC can improve its prognosis. Although the molecular mechanisms of NSCLC development and pathogenesis have been investigated using various histological techniques, the global mortality rate of NSCLC has remained high in recent decades. Therefore, the studies on more effective diagnostic and prognostic markers are ongoing challenges for biomedical research. Thanks to advances in bioinformatics technology, researchers have been able to identify promising biomarkers for NSCLC, and several relevant studies have been published^13,14^.

EMT is the phenomenon in which epithelial cells undergo a phenotypic transformation of fibroblasts or mesenchymal cells, loss of cell polarity, rearrangement of cytoskeleton, and the increased ability of migration and movement^15^. It involves a variety of biological and pathological processes, such as embryonic development, wound healing, cancer cell metastasis, and drug resistance^16^. EMT also plays vital role in the progression of lung cancer. EMT-related markers such as E-cadherin and vimentin are associated with prognosis, tumor lymph node metastasis, and tumor stem marker CD133 expression in lung cancer patients^17,18^. EMT is also associated with EGFR-TKI resistance in lung cancer. In EGFR-mutant lung cancer, EMT inhibits BIM through EMT-inducing transcription factors, ZEB1, and TWIST1, and becomes resistant to EGFR-TKIs^19–21^. In addition, the close relationship between the activation of EMT and inflammatory responses of the tumor microenvironment has also been confirmed, indicating that EMT is a candidate biomarker for NSCLC immunotherapy^22,23^.

In this study, we found a gene associated with tumor metastasis by analyzing the TCGA-LUAD database. The prognostic significance of *GPR87* was then validated in four independent validation cohorts. Functional analysis showed that *GPR87* enhanced migration and invasion of LUAD cells. These results suggest that *GPR87* plays an important role in the metastasis of LUAD.

To investigate the potential clinical roles of *GPR87* expression and methylation in LUAD, we analyzed *GPR87* mRNA expression and methylation data in LUAD patients. We originally found that *GPR87* mRNA was highly expressed in LUAD and that *GPR87* mRNA expression was significantly and negatively correlated with *GPR87* methylation. In addition, we validated the prognostic roles of *GPR87* expression in four independent validation datasets, and these results suggest the potential prognostic values of *GPR87* expression in LUAD patients. Finally, we conducted a meta-analysis of 1639 LUAD patients from five different databases to further prove that *GPR87* expression was an independent prognostic variable for OS in LUAD patients.

The high-risk group will have more Treg cell infiltration. It has been reported in the literature that Treg cells promote tumor expression of more immunosuppressive molecules by suppressing CD8 + T cells, causing immune escape of tumors^24,25^. Tumor-associated macrophages originate from peripheral monocytes, and their tumor-promoting function supports tumor-associated angiogenesis and promotes cancer cell invasion, migration, and vascular metastasis. Tumor-associated macrophages are mainly divided into M1 and M2 subtypes. M1 macrophages located on tumor cell islets are usually associated with a better prognosis, whereas M2 macrophages, which are more abundant in the tumor stroma, are associated with a poorer prognosis^26^. CD4 + T cell subsets, such as Th1, Th2, Th17, and regulatory T (Treg) cells, play a crucial role in cancer immunity. The Th2 subset of CD4 + T cells secretes IL-4, IL-5, and IL-13 and activates B cells to become antibody-secreting plasma cells. Notably, the balance between Th1 and Th2 differentiation is crucial for immune homeostasis, and the shift of Th1/Th2 balance to Th2 cells is associated with immunosuppression and cancer progression^27^. In addition, ALEXANDRA et al. showed that the balance of CD4+ and CD8+ lymphocytes infiltrating the tumor mesenchyme is a crucial factor in determining antitumor immune surveillance and has a solid prognostic value as a predictive marker for immunotherapy in treatable NSCLC. High CD4+/CD8+ ratio defined a worst prognosis^28^. The CD4+/CD8+ ratio was higher in the *GPR87* high-expression group than the low *GPR87* expression group, suggesting that the high-expression group patients were correspondingly poorer for non-small cell lung cancer immunotherapy, consistent with the results of the TIDE algorithm analysis. Our study suggests that the expression level of *GPR87* can be a predictor of immune cell infiltration and immunotherapy in non-small cell lung cancer.

A distinct mode of the immune conditions was further found between the low and high-*GPR87* groups. Our studies suggested that *GPR87* could be used as prognostic markers and indexes of immune status. In addition, The TIDE score of the high-*GPR87* group was higher than that of the low-*GPR87* one. The TIDE score reflects ICI response in LUAD patients. The lower the TIDE score and the more sensitive the patient was to ICIs. Therefore, the low-risk patients might benefit more from immunotherapy, which may be related to the relief of immunosuppression. Current bottlenecks in lung cancer treatment also forced such patients to return to conventional chemotherapy. Therefore, mRNA expression data were utilized to investigate the sensitivity of high- and low-*GPR87* patients to conventional chemotherapeutic agents (ABT.888, ATRA, axitinib, BIRB.0796, CCT007093, EHT.1864, metformin, methotrexate, GDC0941, and AZD8055). These studies suggest that high-*GPR87* patients performed better than low-*GPR87* ones with the same drugs. Our studies demonstrated the sensitivity of patients with high- and low-*GPR87* expression to ten chemotherapeutic agents, providing a therapeutic target for investigators to overcome drug resistance.

However, the current study has several shortcomings that should be considered when interpreting our results. First, transcriptome analysis can only reflect changes in mRNA levels, not overall changes. Second, the mechanism of *GPR87*-induced EMT was to be investigated. Finally, our results need to be validated with in vivo experiments as well as clinical samples.

## Methods

### Data collection and processing

The RNA-seq TPM data of LUAD, containing corresponding clinical data, were acquired from the TCGA^29^, including 497 LUAD and 54 normal tissues. Preprocessed methylation data were downloaded from UCSC XENA databases^30^. The datasets (GSE31210, GSE50081, GSE68465, and GSE30219) of NSCLC with survival data were downloaded from the GEO database as the validation sets^31–33^. GSE31210, GSE50081, and GSE30219 data were obtained from the GPL570 platform (Affymetrix Human Genome U133 Plus 2.0 Array) with 226, 181, and 293 NSCLC tissue samples, respectively. GSE68465 data were obtained from the GPL96 platform (Affymetrix Human Genome U133A Array) with 442 LUAD samples. The relationship between *GPR87* expression and patient STAGE was analyzed using TISIDB^34^.

### Differential expression analysis

DEGs were analyzed using the R software limma package (version 3.46.0) in LUAD and its adjacent normal tissues^35^. The Oncomine database (https://www.oncomine.org/resource/main.html) was used to analyze the expression of *GPR87* between tumor and normal tissues^36^.

### Receiver operating characteristic curve

We used the receiver operating characteristic curve (ROC) to analyze the predictive efficacy of molecular expression and predicted outcomes, the larger the area under the curve, the higher the diagnostic accuracy. We selected tumor and normal as predicted outcomes.

### Single-sample gene set enrichment analysis

The patients were divided into two groups according to the expression of *GPR87*. ssGSEA was used to calculate a metastatic signature score for each sample through the R package GSVA (version 1.38.2). Default parameters in the GSVA package were used^37^. The metastatic gene signatures were obtained from the MSigDB^38^.

### Functional enrichment analysis

The org.Hs.eg.db package was sued to convert the gene symbol into entrezID. GO and KEGG enrichment analyses were then performed using the clusterProfiler package (version 3.18.1)^39^. *P* < 0.05 was considered a statistical significance. The ggplot package was used to draw the diagram of GO and KEGG. The GSEA algorithm was used to identify enriched pathways between the *GPR87* low- and high-expression groups. A ranked gene list was generated by comparing the *GPR87* low- and high-expression groups. GSEA was then used to assess the enrichment of different pathway genomes in this ranked gene list.

### Survival analysis

The relationship between *GPR87* expression and prognosis was analyzed by Kaplan–Meier curves using the survminer package (version 0.4.9) in R software. In addition, we drew a nomogram including the clinical factors and the expression of *GPR87*. The calibration curve was painted to illustrate the accurateness of this model in predicting the survival of LUAD patients.

### Meta-analysis

We assessed the prognostic significance of *GPR87* in LUAD patients using a meta-analysis of five datasets. The combined HR and 95% CI were computed to assess the relationship between *GPR87* expression and the prognosis of LUAD patients. The heterogeneity of the five datasets was evaluated by Q-test (I^2^ statistic). A fixed-effects model was chosen for the combination if there was no significant heterogeneity (I^2^ < 50%). Meta-analysis was performed using the Meta-Package (version 4.18-0) of R software^40^.

### Methylation analysis

The *GPR87* hypo- and hypermethylated groups were classified based on the median *GPR87* DNA methylation levels in the TCGA-LUAD dataset. The correlations between *GPR87* gene expression or DNA methylation and a range of categorical variables were analyzed using the chi-square test or Fisher’s exact test. The spearman analysis was used to investigate the relationship between *GPR87* gene expression and DNA methylation.

### Immunity analysis and gene expression

The differences in immune cell infiltration or immune responses between the *GPR87* high- and low-expression groups were evaluated using the CIBERSORT^41^, ESTIMATE^42^, ssGSEA, and TIMER algorithms^43^. Heatmaps revealed the differences in immune cell infiltration using different algorithms. In addition, we analyzed the differences in the expression levels of immune checkpoints between the 2 groups.

### Evaluation of immunotherapeutic strategies with *GPR87* expression

We predicted potential ICI responses with the TIDE algorithm, which integrated the characteristics of T cell dysfunction and exclusion into the tumor immune escape model to predict the ICI responses^44^.

### Evaluation of the sensitivity of chemotherapeutic agents

The pRRophetic algorithm was applied to predict the IC50 of chemotherapeutic drugs via constructing a ridge regression model based on the expression profiles of cancer drug sensitivity genomics and TCGA gene expression profiles^45^. To explore the effects of *GPR87* on chemotherapy sensitivity in LUAD patients, we used the pRRophetic package (version 0.5) to predict the IC50.

### Cells

LUAD cell lines, A549 and H1299, were purchased from the Type Culture Center of the Chinese Academy of Sciences (Shanghai, China), and cultured in RPMI-1640 medium (HyClone, USA) containing 10% fetal bovine serum. All cells were cultured in a standard tissue culture incubator maintained at 37 °C with 95% humidity and 5% CO_2_. We transfected *GPR87*-specific or non-specific siRNAs synthesized by Beijing TsingKe Company (Beijing, China) using the jetPRIME transfection reagent (Polyplus-transfection^®^ SA, France). siRNA sequences are listed in Table S2.

### RNA extraction and qRT-PCR

Total RNA was isolated from cells using TRIzol reagent (Vazyme, China). We used HiScript^®^ Q RT SuperMix (Vazyme, China) to transcribe RNA and ChamQTM SYBR^®^ qPCR Master Mix (Vazyme, China) for qRT-PCR. The primer sequences are listed in Table S1.

### Flow cytometry

The negative control cells and siRNA-treated cells were collected, added with binding buffer and Annexin V-FITC staining solution, and kept at 4 °C for 15 min in the dark. After 5 min incubation with a propidium iodide solution, cells were analyzed with the CytoFLEX instrument.

### Colony-forming assay

After transfection, 1000 cells per well were seeded in a six-well plate. After 2 weeks, colonies were fixed with 4% paraformaldehyde, stained with 0.5% crystal violet, and counted.

### Wound healing assay

We seeded transfected cells into six-well plates. A pipette tip was used to make a straight scratch line in the cell monolayer. The following formula was used to calculate the migration rate: wound closure rate (%) = (area of initial scratch − cell-free area of final imaging)/distance of initial scratch.

### Modified Boyden chamber assay

A549 or H1299 cells (3 × 10^5^ cells in 200 μL serum-free medium) were seeded in the upper chambers pre-coated with Matrigel (BD). A culture medium (500 μL) with 10% fetal bovine serum was added to the lower chamber. Migrating cells were fixed with formaldehyde 48 h later and stained with 0.5% crystal violet (Sigma-Aldrich; Merck KGaA). Five fields per chamber were randomly observed by microscopy (Olympus, Japan; 200×), and the number of cells in each field was quantified.

### Immunoblotting

The whole-cell proteins were extracted using RIPA lysis buffer (Beyotime, China). SDS-PAGE (10%) gel (EpiZyme, China) was used to separate the proteins, and proteins were transferred to PVDF membranes. We probed *GPR87*, E-cadherin, N-cadherin, Vimentin, and GAPDH with the corresponding antibodies at 4 °C for 12 h and detected them with chemiluminescence 2 h after incubation with HRP-labeled secondary antibodies (Bio-Rad, USA). Antibodies are listed in Table S3.

### Statistics and reproducibility

All experimental data were expressed as mean ± standard deviation (SD). Statistical analysis of variance was conducted by GraphPad Prism 7 and one-way analysis of variance (ANOVA) to identify significant differences among groups. The student *t*-test was used to test for the significance between two groups, and statistical significance was achieved at *P* ≤ 0.05 (ns, *P* ≥ 0.05; *P < 0.05; **P < 0.01; ***P < 0.001). All experiments were taken from distinct samples and the number of biological replicates (3).

### Reporting summary

Further information on research design is available in the Nature Research Reporting Summary linked to this article.

## Supplementary information

Supplementary Information

Description of Additional Supplementary Files

Supplementary Data 1

Supplementary Data 2

Reporting Summary

## Data Availability

All data used in this work can be acquired from the Gene Expression Omnibus (GSE31210, GSE50081, and GSE68465;GSE30219) and the UCSC Xena (https://xenabrowser.net/datapages/?cohort=GDC%20TCGA%20Lung%20Adenocarcinoma%20(LUAD)&removeHub=https%3 A%2 F%2Fxena.treehouse.gi.ucsc.edu%3A443). The data of differentially expressed genes in patients with high and low expression of CARM1 and data for meta-analysis were provided as Supplementary Data 1 and 2. The uncropped gel images were provided in Supplementary Fig. S4. Any remaining information can be obtained from the corresponding author upon reasonable request.
