# Supplementary material for: GPR87 promotes tumor cell invasion and mediates the immunogenomic landscape of lung adenocarcinoma
Source: Commun Biol. 2022 Jul 5;5:663. doi: 10.1038/s42003-022-03506-6 (PMC9256611; doi:10.1038/s42003-022-03506-6)
Supplement: Supplementary file 1 — Supplementary Information [file 42003_2022_3506_MOESM1_ESM.docx]

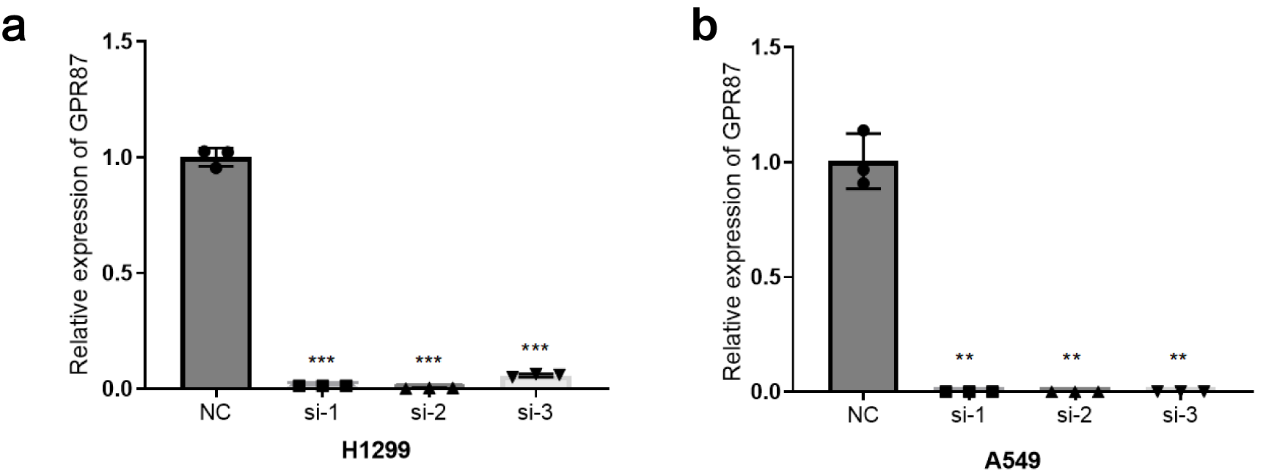


**Fig. S1: siRNA knockdown efficiency validation.** qRT-PCR analysis of GPR87 expression in H1299 (**a**) and A549 (**b**) cells transfected with GPR87 siRNA (KD) and normal controls (NC).


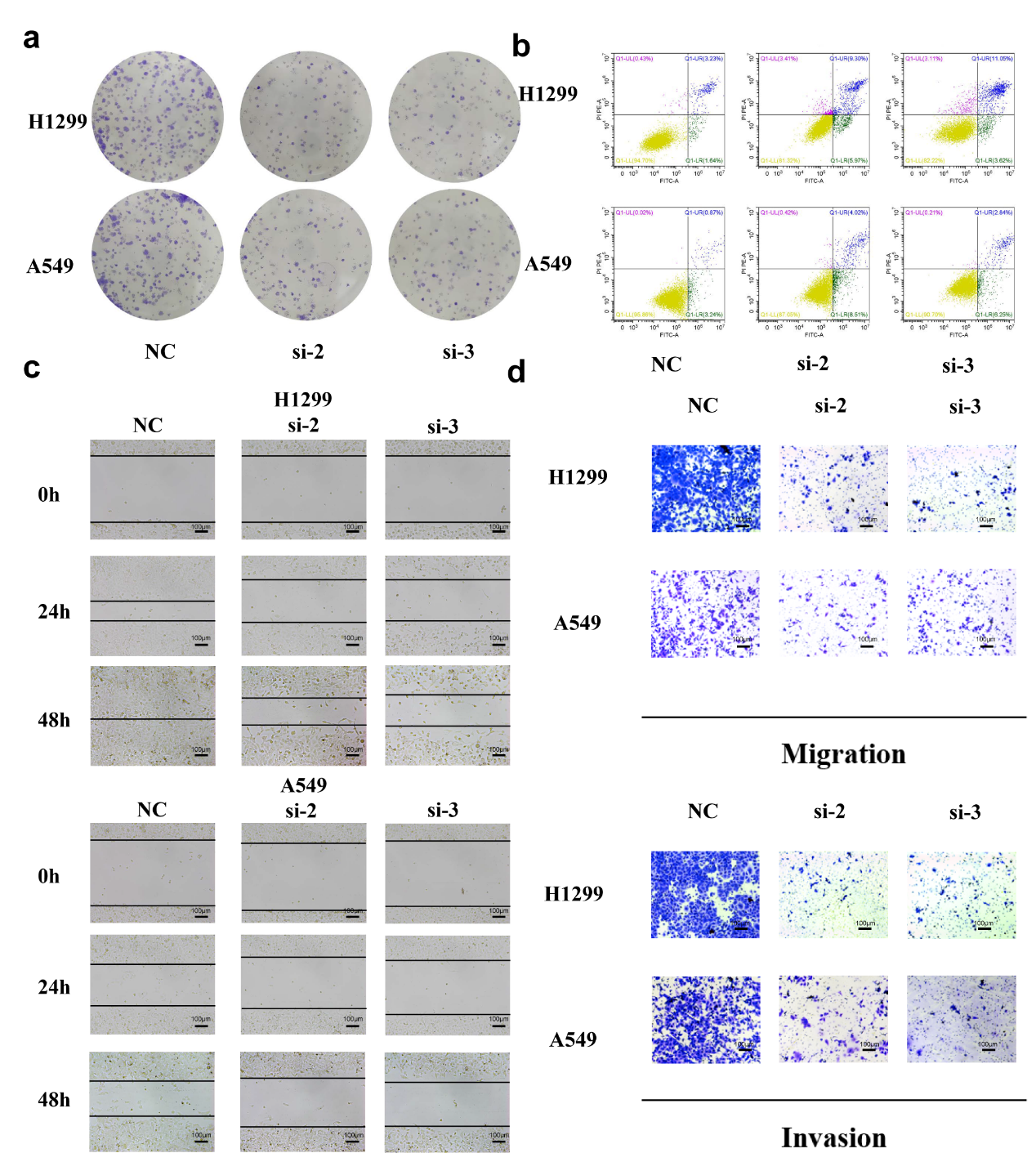


**Fig. S2: GPR87 knockdown inhibits LUAD cell clonogenesis, apoptosis, migration and invasion.** **a** H1299 and A549 cell clonogenesis was detected using clone formation assays. **b** Apoptosis was detected by flow cytometry. **c** Cell migration was evaluated by wound healing assays and reduced by GPR87 knockdown. Scale bars: 100 μm. **d** Migration and invasion of A549 and H1299 cells transfected with GPR87 siRNA were evaluated by modified Boyden chamber assays. Scale bars: 100 μm.


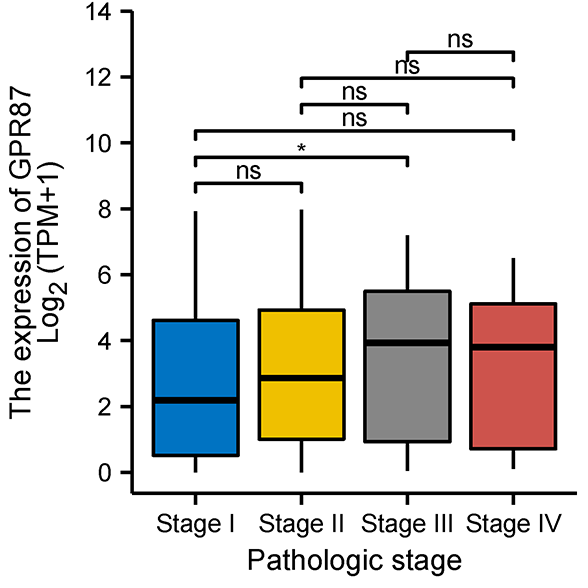


**Fig. S3: The breakdown of GPR87 expression by stages in LUAD.**


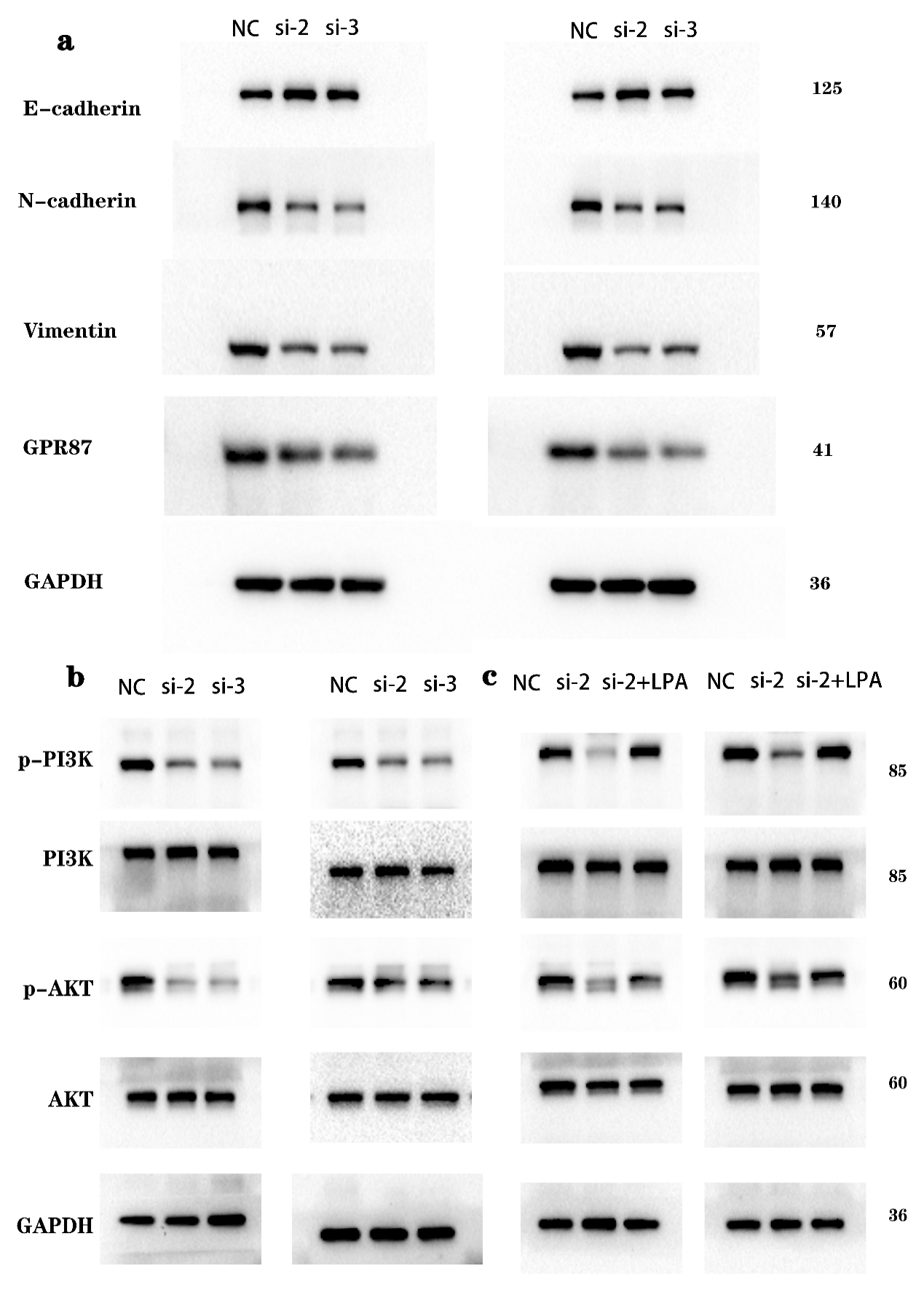


**Fig. S4: Images of original Western blots used in Fig 3.** Fig. S4a corresponds to Fig. 3e. Fig. S4b corresponds to Fig. 3f. Fig. S4c corresponds to Fig. 3g.

Table S1 Primers used for RT-PCR.

| **Gene** | **Forward Primer (5’-3’)** | **Reverse Primer (5’-3’)** |
| --- | --- | --- |
| GPR87 | GAAAATCCACTTCCCTGCCG | AGGAAACACTTGGGGACGAT |
| GAPDH | GGAGCGAGATCCCTCCAAAAT | GGCTGTTGTCATACTTCTCATGG |

Table S2 siRNA sequence.

|  | **sense（5'-3'）** | **antisence（5'-3'）** |
| --- | --- | --- |
| **NC** | UUCUCCGAACGUGUCACGUTT | UAUCUGACAGGGCUUGAGCTT |
| **siRNA-1** | GCUUCAUAUUCUAUCUCAATT | UUGAGAUAGAAUAUGAAGCTT |
| **siRNA-2** | CCAAGAGAGUCACAAUUCATT | UGAAUUGUGACUCUCUUGGTT |
| **siRNA-3** | CCAUAUCACUUGUGCAGAATT | UUCUGCACAAGUGAUAUGGTT |

Table S3 Antibodies used for immunoblotting.

| Target protein | Manufacturer | Catalog number | Dilution |
| --- | --- | --- | --- |
| GAPDH | Proteintech | 10494-1-AP | 1:1000 |
| E-Cadherin | Abclonal | A3044 | 1:1000 |
| N-Cadherin | Abclonal | A19083 | 1:1000 |
| GPR87 | Abclonal | A15162 | 1:1000 |
| Vimentin | Cell signaling technology | 5741 | 1:1000 |
| PI3K | Abclonal | A11526 | 1:1000 |
| P-PI3K | Abclonal | AP0854 | 1:1000 |
| Akt | Cell signaling technology | 4691 | 1:1000 |
| P-Akt | Cell signaling technology | 4060 | 1:2000 |
