# Supplementary material for: GPR87 promotes tumor cell invasion and mediates the immunogenomic landscape of lung adenocarcinoma
Source: Commun Biol. 2022 Jul 5;5:663. doi: 10.1038/s42003-022-03506-6 (PMC9256611; doi:10.1038/s42003-022-03506-6)
Supplement: Supplementary file 2 — Description of Additional Supplementary Files [file 42003_2022_3506_MOESM2_ESM.pdf]

Description of Additional Supplementary Files

File name: Supplementary Data 1
Description: Differentially expressed genes in patients with high and low expression of GPR87.

File name: Supplementary Data 2
Description: Source data for meta analysis.
