# Supplementary material for: GPR87 promotes tumor cell invasion and mediates the immunogenomic landscape of lung adenocarcinoma
Source: Commun Biol. 2022 Jul 5;5:663. doi: 10.1038/s42003-022-03506-6 (PMC9256611; doi:10.1038/s42003-022-03506-6)
Supplement: Supplementary file 5 — Reporting Summary [file 42003_2022_3506_MOESM5_ESM.pdf]

nature research

Corresponding author(s): Conghua Xie

Last updated by author(s): May 7, 2022

Reporting Summary

Nature Research wishes to improve the reproducibility of the work that we publish. This form provides structure for consistency and transparency in reporting. For further information on Nature Research policies, see our Editorial Policies and the Editorial Policy Checklist.

Statistics

For all statistical analyses, confirm that the following items are present in the figure legend, table legend, main text, or Methods section.

n/a	Confirmed	
☐	☒	The exact sample size (n) for each experimental group/condition, given as a discrete number and unit of measurement
☐	☒	A statement on whether measurements were taken from distinct samples or whether the same sample was measured repeatedly
☐	☒	The statistical test(s) used AND whether they are one- or two-sided<br>*Only common tests should be described solely by name; describe more complex techniques in the Methods section.*
☐	☒	A description of all covariates tested
☐	☒	A description of any assumptions or corrections, such as tests of normality and adjustment for multiple comparisons
☐	☒	A full description of the statistical parameters including central tendency (e.g. means) or other basic estimates (e.g. regression coefficient) AND variation (e.g. standard deviation) or associated estimates of uncertainty (e.g. confidence intervals)
☐	☒	For null hypothesis testing, the test statistic (e.g. F, t, r) with confidence intervals, effect sizes, degrees of freedom and P value noted<br>*Give P values as exact values whenever suitable.*
☒	☐	For Bayesian analysis, information on the choice of priors and Markov chain Monte Carlo settings
☒	☐	For hierarchical and complex designs, identification of the appropriate level for tests and full reporting of outcomes
☒	☐	Estimates of effect sizes (e.g. Cohen's d, Pearson's r), indicating how they were calculated

Our web collection on statistics for biologists contains articles on many of the points above.

Software and code

Policy information about availability of computer code

Data collection	No software was used.
Data analysis	No software was used.

For manuscripts utilizing custom algorithms or software that are central to the research but not yet described in published literature, software must be made available to editors and reviewers. We strongly encourage code deposition in a community repository (e.g. GitHub). See the Nature Research guidelines for submitting code & software for further information.

Data

Policy information about availability of data

All manuscripts must include a data availability statement. This statement should provide the following information, where applicable:

- Accession codes, unique identifiers, or web links for publicly available datasets
- A list of figures that have associated raw data
- A description of any restrictions on data availability

The datasets generated during and analysed during the current study are available from the corresponding author on reasonable request.

Field-specific reporting

Please select the one below that is the best fit for your research. If you are not sure, read the appropriate sections before making your selection.

☒ Life sciences ☐ Behavioural & social sciences ☐ Ecological, evolutionary & environmental sciences

For a reference copy of the document with all sections, see nature.com/documents/nr-reporting-summary-flat.pdf

Life sciences study design

All studies must disclose on these points even when the disclosure is negative.

Sample size	No sample size calculation was performed.
Data exclusions	No data were excluded from the analysis.
Replication	All attempts at replication were successful.
Randomization	Not relevent
Blinding	Not relevant

Reporting for specific materials, systems and methods

We require information from authors about some types of materials, experimental systems and methods used in many studies. Here, indicate whether each material, system or method listed is relevant to your study. If you are not sure if a list item applies to your research, read the appropriate section before selecting a response.

Materials & experimental systems

n/a	Involved in the study	
☐	☒	Antibodies
☐	☒	Eukaryotic cell lines
☒	☐	Palaeontology and archaeology
☒	☐	Animals and other organisms
☒	☐	Human research participants
☒	☐	Clinical data
☒	☐	Dual use research of concern

Methods

n/a	Involved in the study	
☒	☐	ChIP-seq
☐	☒	Flow cytometry
☒	☐	MRI-based neuroimaging

Antibodies

Antibodies used	GAPDH,western blot, Proteintech, 10494-1-AP<br>E-Cadherin, western blot, Abclonal, A3044<br>N-Cadherin, western blot, Abclonal, A19083<br>GPR87, western blot, Abclonal, A15162<br>Vimentin, western blot, Abclonal, A19607
Validation	GAPDH; human, mouse, rat, pig, arabidopsis , corn , cabbage, rice; CoIP, IF, IHC, IP, RIP, WB<br>E-Cadherin; Human, Mouse; WB,IHC,IF<br>N-Cadherin; Human, Mouse, Rat; WB,IHC,IF<br>GPR87; Human; WB<br>Vimentin;Human, Mouse, Rat; WB,IHC,IF,IP

Eukaryotic cell lines

Policy information about cell lines

Cell line source(s)	cell bank,type culture collection,chinese academy of sciences
Authentication	The genomic DNA was purified with Purelink@ Genomic DNA Kits in our Bank.The DNA sample was analysed in Beijing Microread GeneticsCo.,Ltd.The sample was amplified withGoldeneyeTM20A STR Complex Amplification Kit.The profiles STR loci and Amelogenin gene were characterized on ABI 3100 TypeGenetic Analysis Instrument.
Mycoplasma contamination	all cell lines tested negative for mycoplasma contamination

Commonly misidentified lines (See ICLAC register) | none

Flow Cytometry

Plots

Confirm that:

- [ ] The axis labels state the marker and fluorochrome used (e.g. CD4-FITC).
- [ ] The axis scales are clearly visible. Include numbers along axes only for bottom left plot of group (a 'group' is an analysis of identical markers).
- [ ] All plots are contour plots with outliers or pseudocolor plots.
- [x] A numerical value for number of cells or percentage (with statistics) is provided.

Methodology

Sample preparation: The negative control cells and siRNA-treated cells were collected, added with binding buffer and Annexin V-FITC staining solution, and kept at 4 °C for 15 minutes in the dark. After 5 min incubation with propidium iodide solution, cells were analyzed with CytoFLEX instrument.

Instrument: CytoFLEX instrument

Software: CytoFLEX

Cell population abundance: To determine the percentage of cell population distribution, we quantified the population of apoptotic cells with fluorescence in the green emission spectrum, necrotic cells with red fluorescence, and late apoptotic cells with both green and red fluorescence.

Gating strategy: *Describe the gating strategy used for all relevant experiments, specifying the preliminary FSC/SSC gates of the starting cell population, indicating where boundaries between "positive" and "negative" staining cell populations are defined.*

- [ ] Tick this box to confirm that a figure exemplifying the gating strategy is provided in the Supplementary Information.
